# Ki67 Labelling Index predicts clinical outcome and survival in oral squamous cell carcinoma

**DOI:** 10.1590/1678-7757-2020-0751

**Published:** 2021-03-01

**Authors:** Amol Ramchandra GADBAIL, Sachin C SARODE, Minal S CHAUDHARY, Shailesh M GONDIVKAR, Satyajit Ashok TEKADE, Monal YUWANATI, Shankargouda PATIL

**Affiliations:** 1 Indira Gandhi Government Medical College and Hospital Department of Dentistry NagpurMaharashtra India Indira Gandhi Government Medical College and Hospital, Department of Dentistry, Nagpur, Maharashtra, India.; 2 Dr. D.Y. Patil Dental College and Hospital Department of Oral Pathology and Microbiology Pimpri India Dr. D.Y. Patil Dental College and Hospital, Dr. D.Y. Patil Vidyapeeth, Sant-Tukaram Nagar, Department of Oral Pathology and Microbiology, Pimpri, India; 3 Datta Meghe Institute of Medical Sciences Sharad Pawar Dental College & Hospital Department of Oral Pathology and Microbiology WardhaMaharashtra India Datta Meghe Institute of Medical Sciences, Sharad Pawar Dental College & Hospital, Department of Oral Pathology and Microbiology, Sawangi (M), Wardha, Maharashtra, India.; 4 Government Dental College & Hospital Department of Oral Medicine and Radiology NagpurMaharashtra India Government Dental College & Hospital, Department of Oral Medicine and Radiology, Nagpur, Maharashtra, India.; 5 Modern Dental College & Research Centre Department of Oral Pathology and Microbiology IndoreMadhya Pradesh India Modern Dental College & Research Centre, Department of Oral Pathology and Microbiology, Gandhi Nagar, Indore, Madhya Pradesh 453112, India.; 6 People’s University People’s College of Dental Science & Research Centre Department of Oral Pathology and Microbiology BhopalMadhya Pradesh India People’s University, People’s College of Dental Science & Research Centre, Department of Oral Pathology and Microbiology, Bhopal, Madhya Pradesh, India.; 7 Jazan University College of Dentistry Department of Maxillofacial Surgery and Diagnostic Sciences Jazan Saudi Arabia Jazan University, College of Dentistry, Division of Oral Pathology, Department of Maxillofacial Surgery and Diagnostic Sciences, Jazan, Saudi Arabia.

**Keywords:** Oral Squamous Cell Carcinoma, Ki67 Labelling Index, Proliferation, Prognosis, Survival

## Abstract

**Objective:**

To investigate the Ki 67 expression and its correlation with clinicopathological features and 3 years as well as 5 years survival rate in oral squamous cell carcinoma (OSCC).

**Methodology:**

Total 217cases of OSCC primarily treated with surgery with or without radiation were included. All patients were followed up for 3 years and 150 were followed up of 5 years for disease free survival. The immunohistochemistry was carried out on neutral buffered formalin fixed paraffin embedded tissue to evaluate the expression of Ki67.

**Results:**

The Ki67 labeling index (LI) was significantly higher with respect to adverse clinicopathological parameters such as histopathological grading (p<0.001), clinical TNM staging (p<0.001) and nodal metastasis (p<0.001). The OSCC patients survived for less than 3 and 5 years were showed significantly higher Ki67 LI as compared to diseases free survived more than 3 and 5 years(p<0.001). The three years survival rate of OSCC patient significantly higher with low Ki67 LI (≤45) 96.2%, followed by moderate Ki67 LI (46 to 60) 60.7% and high Ki67 LI (≥61) 37.7% (p<0.001). The five years survival rate of OSCC patient statistically significantly higher with low Ki67 LI (≤45)93.3%, followed by moderate Ki67 LI (46 to 60) 46.8% and Ki67 LI (≥61) 23.3% (p<0.001).

**Conclusion:**

The measurement of cell proliferative activity by using Ki67 antigen expression in individual OSCC might provide unique, predictive information on clinical outcome, prognosis and deciding treatment modalities in OSCC.

## Introduction

Globally, oral squamous cell carcinoma (OSCC) incidence is 2.7 in 100000 populations. In south-central Asia, the incidence of OSCC cases was highest that is40.9% of all incident cases of OSCC and often associated poor prognosis due to high morbidity and mortality rate.^1^OSCC is extensively investigated for identification and validation of prognostic and predictive markers. However, up until this point, no single marker is consistently acknowledged for routine clinical use in OSCC patients.^2^

Cell proliferation is viewed as a key fundamental biological process in the growth and development as well as upkeep of homeostasis of tissue. The biological behavior of tumor such as local invasion/expansion, local recurrence, metastatic potential as well as disease free survival is predicted by assessment of cell proliferation in histopathology by means of immunohistochemical evaluation.^3^ Thus, evaluation of proliferative status is conventionally viewed as helpful tool in deciding the biological aggressiveness of any tumor.

Ki67 is a large non-histone protein present in the nucleus and nucleolar region, which is seen in cells undergoingproliferation.^4^ Ki67 antigen expression is a reliable and potent biomarker for the accurate, simple and prompt identification of the fraction of cells with proliferative potential in a tumor.^5^ Higher Ki67 expression is an indicator of tumor cells with higher proliferation and locally invasive potential and thus providing one of the best markers for the evaluation of biological aggressiveness of tumor.^6^

Ki67 expression has been accounted to provide a diagnostic and poor prognostic biomarker for OSCC patients.^7-9^ Despite several efforts made in the literature, still there is a controversy existed for Ki-67 being utilized as the proliferation capacity of cancer cells as a predictive biomarker for tumor grade, nodal metastasis, clinical TNM stage and survival rate. Xie, et al.^10^(2016) carried out meta-analysis on prognostic implication of Ki-67 expression in OSCC and recommended further studies on Ki67 expression with five-year survival rate by using standardized immunohistochemistry protocols needed to further strengthen its role as diagnostic and prognostic predictive marker.

Looking at the mandate of five-year survival analysis, the present study was designed to investigate immunohistochemical expression of Ki67 in OSCC with primary focus on three as well as five years survival rate and to assess its predictive value on clinical outcome. These new objectives were investigated on the samples, which were utilized in our previously published papers.^11,12^

## Methodology

The present study was conducted at the Sharad Pawar Dental College and Hospital, Wardha, Maharashtra, India. Institutional ethics committee of Datta Meghe Institute of Medical Sciences, Deemed to be University, Wardha, India was granted the ethical approval for the present study (Ref no. DMIMS (DU)/IEC/2014-15/953, dated 15/12/2014).The written consent was obtained from the patient, who participated in this study.

The study population was retrieved from year 2010 to 2015, which was previously utilized for our research publications^11,12^ with different objectives. The clinically diagnosed and histologically confirmed 217 cases of OSCC, who fulfilled inclusion and exclusion criteria’s, were included. All retrieved cases were primarily treated with wide local surgical excision with normal margins and cervical neck dissection with or without radiation. Patients with coexisting malignancy, previous history of oropharyngeal or oral or any other head and neck cancer, recurrent or distant disease, any other systemic diseases, and pre-operative chemotherapy, radiotherapy, or surgery were excluded from the study. Dissected cervical lymph nodes obtained from surgically excised specimens were examined histopathologically for confirmation of lymph node metastasis. The clinical TNM staging of all OSCC patients were done by using the American Joint Committee of Cancer^13^ clinical TNM staging system for oral and oropharyngeal cancer. The clinical TNM staging was further broadly categories for OSCC cases into early clinical TNM stage (I and II) and advanced clinical TNM stage (III and IV). Demographic details along with clinical features, relevant habit history, and operative details were obtained. Disease free survival of 3 years as well as for some cases 5 years was recorded on follow up.

### Study design

As recommended by Dissanayake, et al.^14^ (2003) neoplastic cells from invasive tumor front region are more informative in studying cell proliferation markers. The histopathological slides of incisional biopsy as well as from resected specimen of OSCC were examined by three oral pathologists independently and those sections showing invasive tumor front of OSCC were utilized for immunohistochemical evaluation of Ki67 antigen expression. All the hematoxylin and eosin stained histopathology and IHC (Ki67) slides were blinded for microscopic analysis. All the included cases were confirmed for histopathological diagnosis of squamous cell carcinoma and were classified in to well-differentiated SCC (WDSCC), moderately-differentiated SCC (MDSCC) and poorly-differentiated SCC (PDSCC). Similarly, three oral pathologists, who were blinded for histopathological grading status of OSCC cases, were independently performed IHC scoring of Ki67. The obtained IHC score for Ki67 were then computed for all OSCC.

### Immunohistochemistry

The standard procedure of immunohistochemistry (IHC) was done for Ki67, by utilizing appropriate controls on neutral buffered formalin fixed paraffin embedded tissue. The tissue sections were treated with 01 mol/L sodium citrate buffer (pH 6.0) in microwave oven for 10 minute followed by bench-cooled for 20 min, and again the same cycle was repeated for the antigen retrieval. Endogenous peroxidase activities of the tissue were blocked by incubating the tissue sections with 3% H2O2 in methanol for 30 minutes. The non-specific tissue reactions were prevented by incubating the tissue sections with 10% serum for 10 minutes. For IHC detection of Ki67 antigen, pre-diluted Monoclonal Mouse Anti-Human, Ki67 antibody [clone MIB-1; Product code:N1633; Dako, Denmark (DD)], was incubated with tissue sections at room temperature in a humidifying chamber for 60 minutes. Known hyperplastic lymph node was used as a positive control for Ki-67. One section from positive control was used as the negative control by omitting the primary antibody and by incubating with serum. The horseradish peroxidase labeled polymer anti-mouse secondary antibody (DakoEnVision System, Product code:K4000, DD) against the primary antibody was incubated at room temperature in humidifying chamber for 30 minutes. To visualized Ki67 antigen antibody complex (expression), the freshly prepared substrate/chromogen solution of 3, 30 Diaminobenzidine (DAB) in provided buffer was used and counterstained in Mayer’s hematoxylin.^15^ The brown coloration of nucleus indicates the positivity for Ki67 antigen expression, where as the blue color of nucleus due to counterstained in Mayer’s hematoxylin indicates the Ki67 negative cells.

### Immunohistochemistry scoring

Neoplastic epithelial cells were viewed as positive for the Ki67 antigen expression, if intranuclear DAB staining (brown color) was observed. Every single stained nuclei were counted positive irrespective of intensity of staining. Invasive tumor front areas as well as areas with highest density of Ki67 labeled neoplastic epithelial cells were located by screening the IHC sections at a 100 X magnification by Leica DM LB2 microscope. In the most heavily Ki67 labeled areas, Ki67 positive neoplastic epithelial cell counts were carried outin 5 randomly selected fields at 400 X magnification. Minimum of 1000Ki67 labeled neoplastic epithelial cells were counted in each section. The number of positively stained nuclei was measured as a percentage of the total number counted neoplastic epithelial cells. Ki67 labeling index (LI) was derived as number positive cells for Ki67 multiplied by 100 and divided by total number observed neoplastic epithelial cells. Based on median and also considering the frequency distribution KI67 LI, cut off values for KI67 LI were generated for three groups to avoid the formation of very small and too many groups.^9^ Thus, based on the KI67 LI, the Ki67 expression were categorized in to low: Ki67 LI≤45, moderate: Ki67 LI 46 to 60 and high: Ki67 LI ≥61.

### Statistical analysis

The data were statistically analyzed using SPSS, version 17.0 for Windows. One-way ANOVA and Tukey’s HSD test were utilized to find out the differences of Ki67 LI amongst the various histopathological grades, clinical TNM stage, and sites of OSCC. Independent student t test was applied to find out the differences of KI67 LI between metastatic and non-metastatic, early and advanced clinical TNM stage, survived and not survived less than 3 year, and survived and not survived less than 5 years. KaplanMeier survival curves as well as the longrank test were used to estimate and compare disease free 3 and 5 years survival with low, moderate and high Ki67 LI. The level of statistical significance was at p<0.05.

## Results

### Details of demographic and clinicopathological features of oral squamous cell carcinoma

Total 217 OSCC patients were included in this study, who were fulfilled the inclusion and exclusion criteria. OSCC cases were predominantly seen in males 159 (73.27%) with male to female 35 (26.72%) ratio was 4.54:1.The age of OSCC patients were ranges from 20 years to 79 years and the mean age of OSCC patient was 50.25 (±12.14) years. The most common site for OSCC was buccal mucosa 84(38.70%) followed by gingivobuccal sulcus 69 (31.79%), tongue 24 (11.05%), retromolar region 16 (7.37%), labial Mucosa 11 (5.06%), palate 10 (4.60%) and floor of mouth 3 (1.38%). Tobacco lime and betel nut (TLB) 135 (62.21%) was the most common habit noted in OSCC patient followed by tobacco lime (TL) 45 (20.73%) and Betel nut (BN) 33 (15.20%). Nearly 95% cases of OSCC were MDSCC 106 (48.84%) and WDSCC 100 (46.08%). MDSCC were the most common histopathological type of OSCC. PDSCC were found in 11 (5.06%) cases of OSCC. Clinical TNM stage IV was the most commonly observed in 94 (43.31%) OSCC followed by Stage III 55 (25.34%) to stage II 52 (23.96%) and stage I 16 (7.37%). Advanced clinical TNM stage was observed in 149 (68.66%) OSCC and early clinical TNM stage was observed in 68 (31.33%) OSCC. The cervical lymph node metastasis was found in 68 (31.33%) OSCC cases. All 217 cases were followed up for 3 years and 148 (68.20%) cases of OSCC were survived. A five year follow up data of 150 cases were available and 81 (54.00%) OSCC cases found to be survived (Table 1).

**Table 1:** Details of demographic and clinicopathological parameters of oral squamous cell carcinoma patients

Characteristics		OSCC (n = 217)
Age	Mean (SD)	50.25 (±12.14)
	Range	20 - 79
Gender	Male	159 (73.27%)
	Female	35 (26.72%)
Site	Buccal Mucosa	84 (38.70%)
	Gingivo-buccal Sulcus	69 (31.79%)
	Tongue	24 (11.05%)
	Retromaolar	16 (7.37%)
	Palate	10 (4.60%)
	Labial Mucosa	11 (5.06%)
	Floor of Mouth	3 (1.38%)
Histopathological diagnosis	WDSCC	100 (46.08%)
	MDSCC	106 (48.84%)
	PDSCC	11 (5.06%)
Habits	Tobacco and lime	45 (20.73%)
	Tobacco lime and Betel nut	135 (62.21%)
	Betel nut	33 (15.20%)
	No habits	4 (1.84%)
Clinical TNM Stage	Stage I	16 (7.37%)
	Stage II	52 (23.96%)
	Stage III	55 (25.34%)
	Stage IV	94 (43.31%)
Clinical TNM Stage	Early Stage	68 (31.33%)
	Advanced Stage	149 (68.66%)
Metastasis	Non-meta	141 (64.97%)
	Metastatic	76 (35.02%)
3 years disease free survival (n=217)	Non-survived	69 (31.79%)
	Survived	148 (68.20%)
5 years disease free survival (n=150)	Non-survived	69 (46.00%)
	Survived	81 (54.00%)

Note:- OSCC: Oral squamous cell carcinoma, WDSCC: Well differentiated squamous cell carcinoma, MDSCC: Moderately differentiated squamous cell carcinoma, PDSCC: Poorly differentiated squamous cell carcinoma, SD: standard deviation, TNM: Tumor (T) Nodes (N) and Metastasis (M).

### Association of Ki67 LI with clinicopathological parameters of oral squamous cell carcinoma

**Figure 1:**
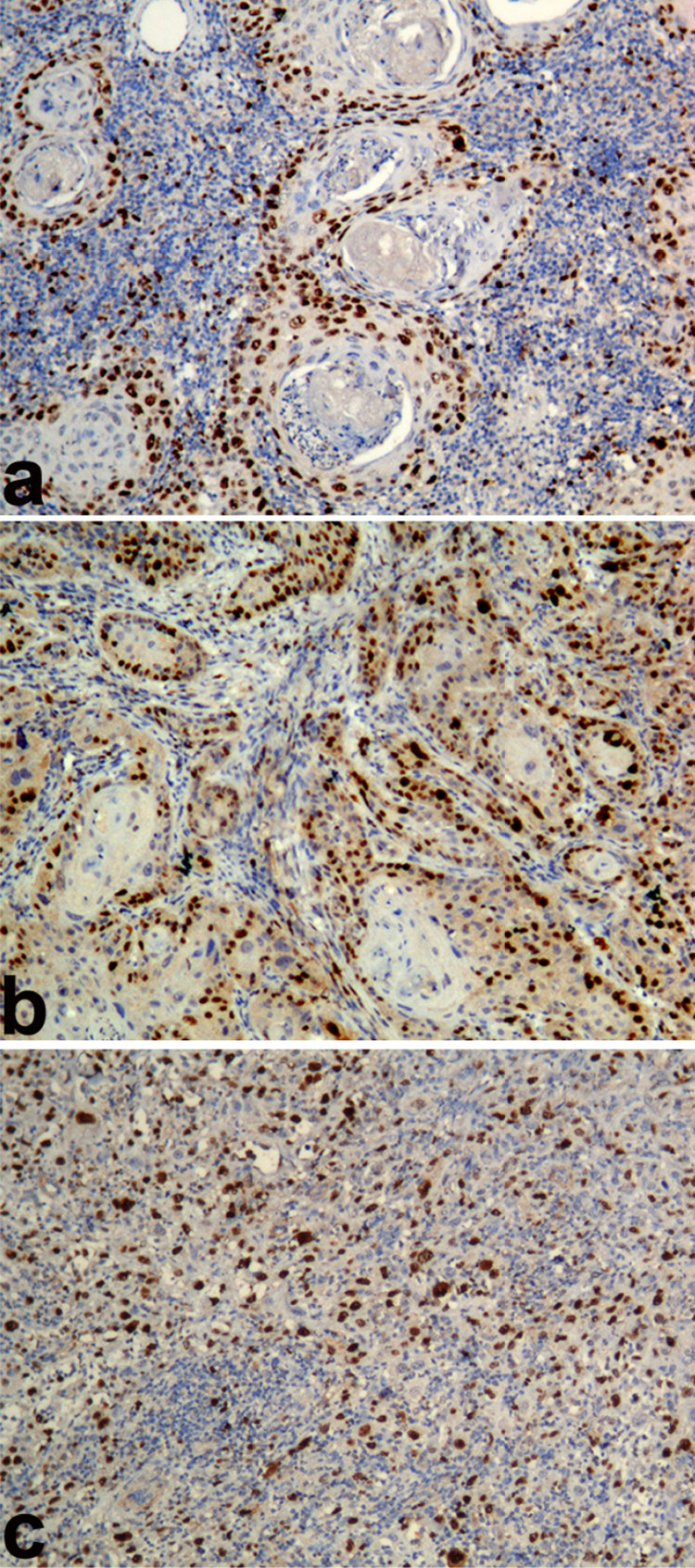
Photomicrograph showing Ki67 antigen expression (Ki67 labeling index) in a) Well differentiated squamous cell carcinoma, b) Moderately differentiated squamous cell carcinoma, c) Poorly Moderately differentiated squamous cell carcinoma (Immunohistochemistry; Magnification X100)

Statistically significant variations of mean Ki67 LI were found among all groups of OSCC (p<0.001). Ki67 LI were observed statistically significantly in ascending order from WDSCC 43.72 (±5.28) to MDSCC 59.41 (±9.75) to PDSCC 74.59 (±9.20) (p<0.001) (Figure 1) (Table 2).

**Table 2:** Comparative analysis of Ki67 labeling index in clinicopathological and survival of all Oral squamous cell carcinoma

Parameters	Groups	N	Mean	Sta. Dev.	Tukey HSD test/ Independent Samples T Test
Histopathological Grading	WDSCC (A)	100	43,72	5,28	A<B<C P<0.001
	MDSCC (B)	106	59,41	9,75	
	PDSCC (C)	11	74,59	9,2	
TNM Stage	Stage I (A)	16	46,54	9,35	A<B (p=0.936); B<C (p=0.433); A<C (p=0.370); C<D (p=0.034); A<D (p=0.004); B<D (p<0.001)
	Stage II (B)	52	48,47	12,99	
	Stage III (C)	55	51,83	10,15	
	Stage IV (D)	94	57,17	11,71	
	Early Stage	68	48,01	12,19	P<0.001
	Advanced Stage	149	55,2	11,42	
Cervical lymph node metastasis	Non-metastatic	141	49,03	10,83	P<0.001
	Metastatic	76	60,23	10,99	
3 years disease free survival (n=217)	Non-survived	69	60,82	10,64	P<0.001
	Survived	148	49,28	10,97	
5 years disease free survival (n=150)	Non-survived	69	60,82	10,64	P<0.001
	Survived	80	49,25	10,2	
Site	Buccal Mucosa	84	52,04	12,34	One-Way ANOVA P=0.374
	GB Sulcus	69	53,74	11,82	
	Tongue	24	53,5	14,43	
	Retromolar	16	55,89	6,64	
	Palate	10	56,28	13,89	
	Labial Mucosa	11	45,79	9,66	
	Floor of Mouth	3	55,28	15,09	

Note:- OSCC: Oral squamous cell carcinoma, WDSCC: Well differentiated squamous cell carcinoma, MDSCC: Moderately differentiated squamous cell carcinoma, PDSCC: Poorly differentiated squamous cell carcinoma, TNM: Tumor (T) Nodes (N) and Metastasis (M).

Statistically significant variations of mean Ki67 LI were found among clinical TNM Stage I, Stage II, Stage III, and Stage IV of OSCC (p<0.001). There was statistical significant difference for Ki67 LI observed between Stage III 51.83 (±10.15) and Stage IV 57.17 (±11.71) (p=0.034); Stage I 46.54 (±9.35) and Stage IV 57.17 (±11.71) (p=0.004); Stage II 48.47 (±12.99) and Stage IV 57.17 (±11.71) (p<0.001). The mean of Ki67 LI were statistically significantly higher in Advanced TNM stage55.20 (±11.42) as compared to Early Stage48.01 (±12.19) of OSCC (p<0.001) (Table 2).

There was no statistical significant variations of mean of Ki67 LI found among various sites of OSCC (P=0.374). However, the Ki67 LI was marginally higher in Palate 56.28 (±13.89) followed by Floor of mouth 55.28 (±15.09), Retromolar 55.89 (±6.64), Gingivo-buccal Sulcus 53.74 (±11.82), Tongue 53.50 (±14.43), Buccal Mucosa 52.04 (±12.34) and Labial Mucosa 45.79 (±9.66) (Table 2).

The mean of Ki67 LI were statistically significantly higher in Metastatic 60.23 (±10.99) as compared to non-metastatic 49.03 (±10.83) cases of OSCC (p<0.001).

### The relationship between Ki67 LI with 3 and 5 years disease free survival of oral squamous cell carcinoma patients

On 3 years of follow up of for disease free survival, the mean of Ki67 LI were statistically significantly higher in Non-survived 60.82 (±10.64) as compared to survived cases 49.28 (±10.97) of OSCC (p<0.001). Similarly on 5 years of follow up of for disease free survival, the mean of Ki67 LI were statistically significantly higher in Non-survived 60.82 (±10.64) as compared to survived cases 49.25 (±10.20) of OSCC (p<0.001) (Table 2).

**Figure 2:**
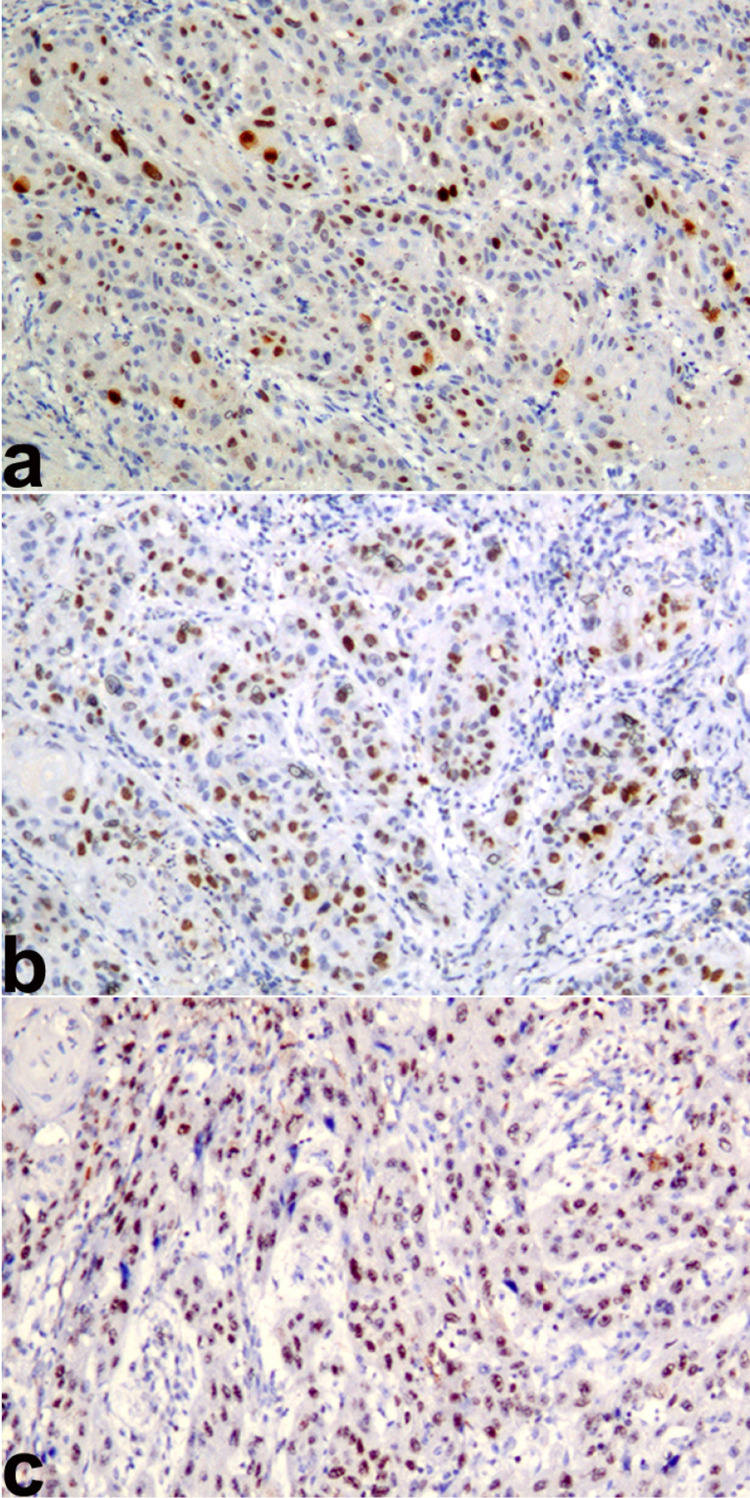
Photomicrograph showing Ki67 antigen expression (Ki67 labeling index) in oral squamous cell carcinoma a) Low Ki67 labeling index (≤45), b) Moderate Ki67 labeling index (46 to 60) and c) High Ki67 labeling index (≥61). (Immunohistochemistry; Magnification X100)

**Figure 3:**
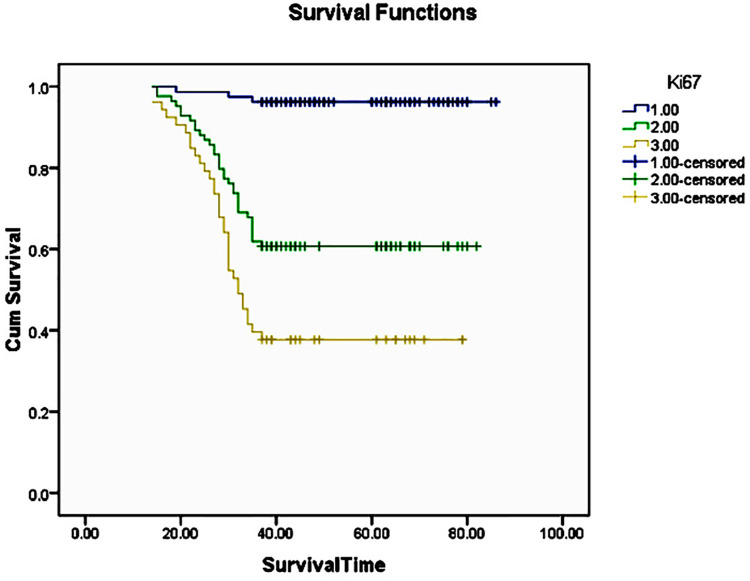
The Kaplan-Meier curve for 3 years disease free survival rate for oral squamous cell carcinoma with respect to 1: Low Ki67 labeling index (≤45), 2: Moderate Ki67 labeling index (46 to 60) and 3: High Ki67 labeling index (≥61)

**Figure 4:**
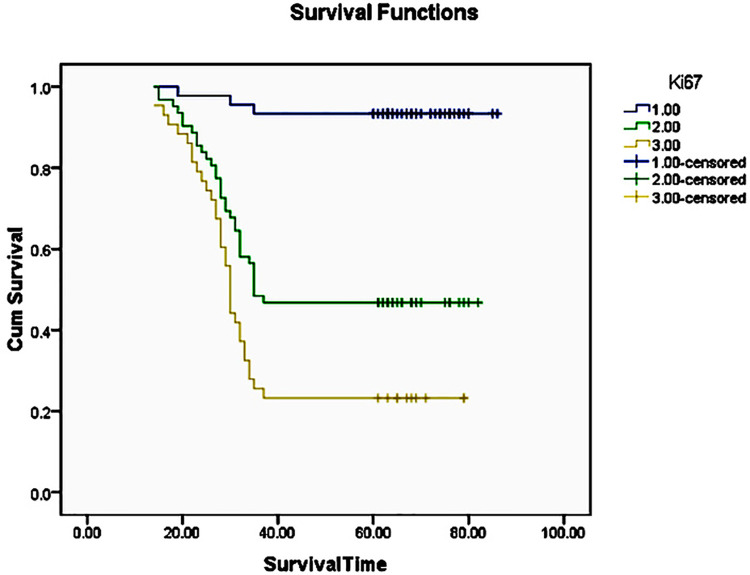
The Kaplan-Meier curve for 5 years disease free survival rate for oral squamous cell carcinoma with respect to 1: Low Ki67 labeling index (≤45), 2: Moderate Ki67 labeling index (46 to 60) and 3: High Ki67 labeling index (≥61)

The three years survival rate of OSCC patient statistically significantly higher with Ki67 LI ≤45(96.2%), followed by Ki67 LI of 46 to 60 (60.7%) and Ki67 LI of ≥61(37.7%) (p<0.001) (Figure 2) (Figure 3). The five years survival rate of OSCC patient statistically significantly higher with Ki67 LI of ≤45 (93.3%), followed by Ki67 LI of 46 to 60 (46.8%) and Ki67 LI of ≥61 (23.3%) (p<0.001) (Figure 4) (Table 3).

**Table 3:** Kaplan Meier Survival analysis: Ki67 labeling index and 3 years and 5 years survival analysis of oral squamous cell carcinoma

Survival	Ki67 labeling index	N	Deaths (N)	Survived (N)	Log Rank (Mantel-Cox)	Breslow (Generalized Wilcoxon)	Tarone-Ware
3 years disease free survival (n=217)	Low (≤45)	80	3	77 (96.2%)	Chi-Square = 55.72; P<0.001	Chi-Square = 53.86; P<0.001	Chi-Square = 54.93; P<0.001
	Moderate (46 to 60)	84	33	51 (60.7%)			
	High (≥61)	53	33	20 (37.7%)			
	Overall	217	69	148 (68.2%)			
5 years disease free survival (n=150)	Low (≤45)	45	3	42 (93.3%)	Chi-Square = 44.94; P<0.001	Chi-Square = 41.38; P<0.001	Chi-Square = 43.38; P<0.001
	Moderate (46 to 60)	62	33	29 (46.8%)			
	High (≥61)	43	33	10 (23.3%)			
	Overall	150	69	81 (54.0%)			

## Discussion

OSCC is an aggressive neoplasm with unpredictable biological behavior. This results in unfavorable prognosis leading to functional impairment as well as high mortality rate irrespective of type of treatment modality rendered.^16^ Treatment modalities and prognosis for OSCC were routinely determined based on clinical features such as tumor size, cervical metastasis to lymph node, distant metastasis and histopathological features mainly histological grade with other characteristics such as mitotic activity, vascular or perineural invasion.^17^ Recently, numerous studies observed that the clinico pathological factors such as advanced age, lymph node metastasis, advanced TNM stage, advanced histopathological grade, the presence of perineural invasion, vascular invasion, tumour–stroma ratio (TSR) and tumour budding were predicator of poor survival in OSCC patients.^18-22^Despite of their claimed usefulness in various studies, characterization of OSCC that are at higher risk for recurrence and relapse is major concern. However, use of cost effective, easy and widely use immunohistochemistry to assess molecular markers leads to an increased understanding of biological behavior of OSSC. With the use of molecular markers predicting biological aggressiveness, locoregional recurrence and survival are useful in identification of patients, who needs more individualized intensive treatment regimes such as chemotherapy and radiotherapy in addition to surgery. An increasing number of studies, have revealed that Ki67 as a reliable cell proliferation biomarker was unregulated in numerous tumors and may be used as an important factor in cancer grading and prognostic evaluation.^23^ Thus, in this study we evaluated Ki67 expression in invasive front region of OSCC with a lager cohort of 217 cases and K67 expression was also correlated with clinical outcome and three as well as five years disease free survival.

In this study, we assess the cut of range value for Ki67 LI as low, moderate and high with respect to three and five years disease free survival of OSCC patients. The three year disease free survival rate of OSCC patient significantly higher with low Ki67 LI (96.2%), followed by moderate KI67 LI (60.7%) and high Ki67 LI (37.7%). The five year disease free survival rate of OSCC patient significantly higher with low Ki67 LI (93.3%), followed by moderate Ki67 LI (46.8%) and high Ki67 LI (23.3%). In this study for the first time we introduce the range value for ki67 LI in OSCC that could be use as prognostic indicator for survival and to decide the treatment regime.

The mean of Ki-67 LI, was significantly higher in OSCC patients survived for less than 3 years as compared to diseases free survived OSCC patients more than 3 years. Similarly, the mean of Ki-67 LI, was significantly higher in OSCC patients survived for less than 5 years as compared to diseases free survived OSCC patients more than 5 years. It has been noticed in previous studies^9,24-26^ that K67 expression in OSCC was identified as prognostic factor for survival rate. However, several other studies^27-30^ have described no association between higher cell proliferation and survival. Recently, Jing, et al.^9^ (2019) studied Ki67 expression in large cohort of 298 OSCC and found that Ki67 expression was independent prognostic marker in OSCC patients. Ki67 expression was also found to be prognostic marker in oropharyngeal and OSCC with a lager cohort of 239 cases.^26^ Therefore, it is summarize that the increase in Ki67 expression associated with a poor prognosis of OSCC. It was also suggested that Ki67 is an indicator for treatment failure in OSCC.^9,26^ Thus, it was further strengthen that the high cell proliferation identified by Ki67 antigen expression is a reliable prognostic marker in oral cancer.

In this study, a significant increase in Ki-67 LI was observed from WDSCC to MDSCC to PDSCC. This results was in agreement with previous studies^9,31-35^ and revealed that as the Ki67 LI increases with poorer the degree of tumor cell differentiation. In contrast, few studies^29,36^ failed to show correlation of Ki67 LI and degree of differentiation. Disparity in the methodology, assessment of Ki67 immunoreactivity, and sample size may result in these conflicting results. Higher expression of Ki67 protein was discovered in undifferentiated/poorly differentiated squamous cells carcinoma. Therefore, results of the present study further strengthen the fact that, the Ki67 LI could be considered as useful potential biomarker in grading the OSCC.

The mean of Ki-67 LI was significantly higher in metastatic as compared to non-metastatic OSCC. Other studies^3,25,32,36^ found insignificant correlation with cell proliferation and cervical lymph node metastasis, which are not in accordance with this study results. In this study large cohort of OSCC was used and results are in accordance with previous studies.^9,28,33,37,38^ This finding may implicate that tumors with high cell proliferation index may point out towards aggressive behavior of a tumour and a potential for metastasis.

In the present study, Ki67 LI was significantly higher in TNM stage IV as compared to stage III. However, non-significant difference of ki67 LI was found amongst stage I, stage II and stage III. The previous studies^3,30,32,34,36^ showed no significant association between Ki67LI and clinical TNM staging. Interestingly, on broadly categories TNM staging, the mean Ki67 LI was significantly higher in advanced stage OSCC as compared to early stage OSCC. These findings are in agreement with previous studies^28,33^ suggesting that the increased Ki67 LI indicates the higher intrinsic growth potential of the tumor resulting in advance TNM staging.

In accordance with previous studies^37,38^ the result of this study have also showed non-significant difference of Ki67 LI amongst site of OSCC.

In this study the Ki67 LI was significantly higher with respect to adverse clinicopathological parameters such as histopathological grading, clinical TNM staging, cervical lymph node metastasis and 3 as well as 5 years disease free survival of OSCC. Thus, it can be suggested that assessment of cell proliferative activity by quantitative measurement of Ki67 positive neoplastic epithelial cells in individual invasive OSCC, may thus reveal unique, predictive information on clinical outcome, prognosis and deciding treatment modalities. Nevertheless, taking into consideration the extensive heterogeneity of tumors, it is recommended further research with standardized uniform immunohistochemistry protocol for Ki67 expression and large cohort of OSCC with 5 to 10 years follow up data to validate the range of Ki67LI for prognosis and treatment outcome.
